# Pulmonary Arterial Hypertension Associated with Portal Hypertension and HIV Infection: Comparative Characteristics and Prognostic Predictors

**DOI:** 10.3390/jcm12103425

**Published:** 2023-05-12

**Authors:** Fabio Dardi, Daniele Guarino, Francesco Cennerazzo, Alberto Ballerini, Ilenia Magnani, Riccardo Bertozzi, Federico Donato, Giulia Martini, Alessandra Manes, Nazzareno Galiè, Massimiliano Palazzini

**Affiliations:** 1 Cardiology Unit of IRCCS Azienda Ospedaliero-Universitaria di Bologna, 40138 Bologna, Italy; 2 Dipartimento DIMEC (Dipartimento di Scienze Mediche e Chirurgiche), Università di Bologna, 40126 Bologna, Italy

**Keywords:** pulmonary arterial hypertension, human immunodeficiency virus (HIV), portal hypertension, Child–Turcotte–Pugh (CTP), model for end-stage liver disease-Na (MELD-Na), highly active antiretroviral therapy (HAART)

## Abstract

Background: Pulmonary arterial hypertension (PAH) may complicate both portal hypertension (Po-PAH) and HIV infection (HIV-PAH). These two conditions, however, frequently coexist in the same patient (HIV/Po-PAH). We evaluated clinical, functional, hemodynamic characteristics and prognostic parameters of these three groups of patients. Methods: We included patients with Po-PAH, HIV-PAH and HIV/Po-PAH referred to a single center. We compared clinical, functional and hemodynamic parameters, severity of liver disease [Child–Turcotte–Pugh (CTP) and Model for End-stage Liver Disease-Na (MELD-Na) scores], CD4 count and highly active antiretroviral therapy (HAART) administration. Prognostic variables were identified through Cox-regression analysis. Results: Patients with Po-PAH (*n* = 128) were the oldest, patients with HIV-PAH (*n* = 41) had the worst hemodynamic profile and patients with HIV/Po-PAH (*n* = 35) had the best exercise capacity. Independent predictors of mortality were age and CTP score for Po-PAH, HAART administration for HIV-PAH, MELD-Na score and hepatic venous-portal gradient for HIV/Po-PAH. Conclusions: Patients with HIV/Po-PAH are younger and have a better exercise capacity than patients with Po-PAH, have a better exercise capacity and hemodynamic profile compared to patients with HIV-PAH, and their prognosis seems to be related to the hepatic disease rather than to HIV infection. The prognosis of patients with Po-PAH and HIV-PAH seems to be related to the underlying disease.

## 1. Introduction

Porto-pulmonary hypertension (Po-PAH) is defined as the association between pulmonary arterial hypertension (PAH) and portal hypertension regardless of the presence of liver cirrhosis. PAH can develop in up to 2–6% of patients with portal hypertension and is currently defined by an increase in mean pulmonary artery pressure (mPAP) > 20 mmHg and in pulmonary vascular resistance (PVR) > 2 WU [1]. Po-PAH carries a poor prognosis and poses significant challenges in the management of patients that require orthotopic liver transplantation (OLT), as it greatly increases perioperative mortality [2,3,4]. PAH-specific therapies have proven to be able to improve hemodynamics, functional status and exercise performance of Po-PAH patients and may contribute in reducing perioperative complications of OLT [5,6,7,8,9,10,11,12]. Complete regression of PAH has been described after OLT, underlying the important role of portal hypertension in the pathogenesis of PAH [13,14] and of OLT in the treatment of this condition [15]. Due to the overlap of disease risk factors, portal hypertension may coexist with human immunodeficiency virus (HIV) infection (i.e., portal hypertension associated with viral chronic liver disease and coexisting HIV infection). In these cases, PAH may result from the combination of both factors (HIV/Po-PAH) and data from the literature regarding the phenotyping and the prognostic parameters of this specific patient subset are lacking. Moreover, after the introduction of highly active antiretroviral therapy (HAART), the survival of HIV-PAH patients has significantly improved but PAH has been described as an important prognostic factor in the survival of HIV patients [16]. Thus, defining the predictors of survival of patients with HIV/Po-PAH may be of relevance in the long-term clinical management of these patients.

The aim of the study was to compare baseline clinical, functional and hemodynamic characteristics of patients affected by Po-PAH, HIV-PAH and HIV/Po-PAH and to identify the main determinants of prognosis of these three groups of patients.

## 2. Materials and Methods

### 2.1. Population

The study was conducted according to the guidelines of the Declaration of Helsinki [17] and was conducted within the context of regular care. Data from all consecutive patients with Po-PAH, HIV-PAH and HIV/Po-PAH who were referred to the Pulmonary Vascular Disease Centre of the Bologna University were included in a prospective electronic registry (ARCA) approved by the Ethics Committee of the St. Orsola-Malpighi Hospital (109/2016/U/Oss). Patient data were pseudonymized and the patients, or their legally authorized representative, provided written informed consent for their use. PAH was diagnosed considering a cut-off of 25 mmHg for mean pulmonary artery pressure (mPAP) and of 3 WU for PVR together with the detection of HIV infection on serological examinations and/or an increase in the hepatic venous-portal gradient (HVPG) > 5 mmHg [18] or the combination of signs suggestive for portal hypertension (including the presence of splenomegaly, thrombocytopenia and/or esophageal varices, or clinical signs of portosystemic shunt). The observation period was from March 1993 to December 2021.

### 2.2. Assessment

Non-invasive and invasive parameters were collected at baseline including the determination of World Health Organization functional class (WHO-FC), 6-min walk distance (6MWD), brain natriuretic peptide (BNP) or N-terminal pro-hormone BNP (NT-proBNP) [the values of these two laboratory tests were divided into three categories (low, intermediate and high) according to the cut-offs of the ESC/ERS guidelines derived risk table] [19] and hemodynamic parameters via right heart catheterization (RHC). The parameters collected through RHC were right atrial pressure (RAP), mPAP, cardiac index (CI), PVR, mixed venous oxygen saturation (SvO_2_) and HVPG. Risk stratification was assessed according to the multiparametric simplified model proposed in our center and PAH-specific sequential combination therapy was indicated according to a goal-oriented treatment strategy if treatment goals were not met [19,20]. We also performed the risk assessment according to the Comparative, Prospective Registry of Newly Initiated Therapies for Pulmonary Hypertension (COMPERA) risk tool [patients were categorized as being at low, intermediate, or high risk by assigning a grade (low = 1, intermediate = 2, high = 3) according to thresholds prescribed by the 2015 ESC/ERS guidelines to the following variables: WHO FC, 6MWD, BNP or NT-pro-BNP, RAP, CI and SvO_2_; the overall risk category is determined by computing the mean of the risk grades from available variables for each patient and rounding to the nearest integer] [21] and the French Pulmonary Hypertension Registry (FPHR) strategy [WHO FC, 6MWD, RAP and CI were considered for each patient and risk is defined by how many low-risk values, according to thresholds prescribed by the 2015 ESC/ERS guidelines, are assigned to a patient; the FPHR methodology defined only the low-risk group but, as suggested in previous works, we considered the presence of three or four low-risk variables to be low risk, the presence of one or two low-risk variables to be intermediate risk, and no low-risk variables to be high risk] [22,23].

In patients affected by Po-PAH and HIV/Po-PAH, we also evaluated at baseline the severity of the liver disease using the Child–Turcotte–Pugh (CTP) and Model for End-stage Liver Disease-Na (MELD-Na) scores. In patients affected by HIV-PAH and HIV/Po-PAH, we collected the absolute value (*n*/mmc) of CD4+ lymphocytes at baseline and whether they were taking HAART at baseline and during follow-up.

### 2.3. Statistics

Baseline variables are presented as *n* (%) for categorical data, and medians (interquartile range) for the continuous data. The multiple imputation approach was used to overcome missing data. Patient characteristics were compared using Pearson’s χ^2^ test or Fisher’s exact test for categorical variables (applying Bonferroni correction for multiple pairwise comparisons). Continuous variables were compared using the Dunn test with Bonferroni correction. We considered *p*-values < 0.05 to be statistically significant. Univariate analysis was performed for all parameters to assess their relation to survival and parameters with a *p*-value < 0.1 were included in the multivariate analysis using the Cox proportional risk model (Stepwise method). Variables with a *p*-value < 0.05 were considered to be independently related to prognosis.

All-cause death was considered; survival was displayed using the Kaplan–Meier plots and the difference between subgroups was tested for significance using the Log-rank test. Patients lost to follow-up or those undergoing lung transplantation were censored as alive at the time of last contact/lung transplantation. For the survival analysis, the date of baseline RHC was used as the starting point to determine length of survival, rather than the date of the PAH diagnosis, to avoid immortal time bias. Statistical analyses were performed using STATA/SE V.15.1 (StataCorp, College Station, TX, USA).

## 3. Results

### 3.1. Patients

A total of 204 patients were eligible for inclusion, of which 128 had Po-PAH, 41 had HIV-PAH and 35 had HIV/Po-PAH. The baseline patient characteristics are shown in Table 1.

### 3.2. Po-PAH

The median follow-up of Po-PAH patients was 48 (17–90) months. During the follow-up 87 patients died (68%). The results of the univariate analysis are shown in Table 2. At the multivariate analysis the only parameters independently associated with survival were age and CTP score (Table 3). The etiology of portal hypertension was alcohol abuse in 26 patients (20%), viral hepatitis in 35 patients (27%), mixed viral and alcohol abuse in 32 patients (25%), autoimmune in 6 patients (5%), cryptogenic in 10 patients (8%), extrahepatic portal vein thrombosis/hypoplasia/malformations in 15 patients (12%), Budd-Chiari syndrome in 1 patient (1%), and other forms of portal hypertension in 3 patients (2%). Thirteen patients underwent liver transplantation during the follow-up. Survival rates were 86% (95% CI 78–91%), 66% (95% CI 57–74%), 51% (95% CI 41–59%), and 27% (95% CI 19–36%) at 1, 3, 5 and 10 years, respectively.

### 3.3. HIV-PAH

The median follow-up of HIV-PAH patients was 64 (25–174) months. During the follow-up 25 patients (61%) died, while 2 patients (4.9%) were lost at follow-up. The results of the univariate analysis are shown in Table 4. At the multivariate analysis, the only parameter independently associated with survival was HAART therapy prescription during follow-up [HR with 95% CI = 0.283 (0.119–0.675); *p*-value = 0.004]. Survival rates were 88% (95% CI 73–95%), 68% (95% CI 51–80%), 55% (95% CI 38–69%), and 44% (95% CI 28–59%) at 1, 3, 5 and 10 years, respectively.

### 3.4. HIV/Po-PAH

The median follow-up of HIV/Po-PAH patients was 54 (24–166) months. During the follow-up, 17 patients died (48.6%) while 1 (2.9%) was lost at follow-up. The results of the univariate analysis are shown in Table 5. At the multivariate analysis, the only parameters independently associated with survival were the MELD-Na score and HVPG (Table 6). The etiology of portal hypertension was alcohol abuse in 1 patient (3%), viral hepatitis in 29 patients (83%), mixed viral and alcohol abuse in 4 patients (11%), and Budd–Chiari syndrome in 1 patient (3%). No patient underwent liver transplantation during the follow-up. Survival rates were 86% (95% CI 69–94%), 67% (95% CI 48–80%), 54% (95% CI 35–69%) and 50 (95% CI 31–66%) at 1, 3, 5 and 10 years, respectively.

### 3.5. Survival

**Figure 1:**
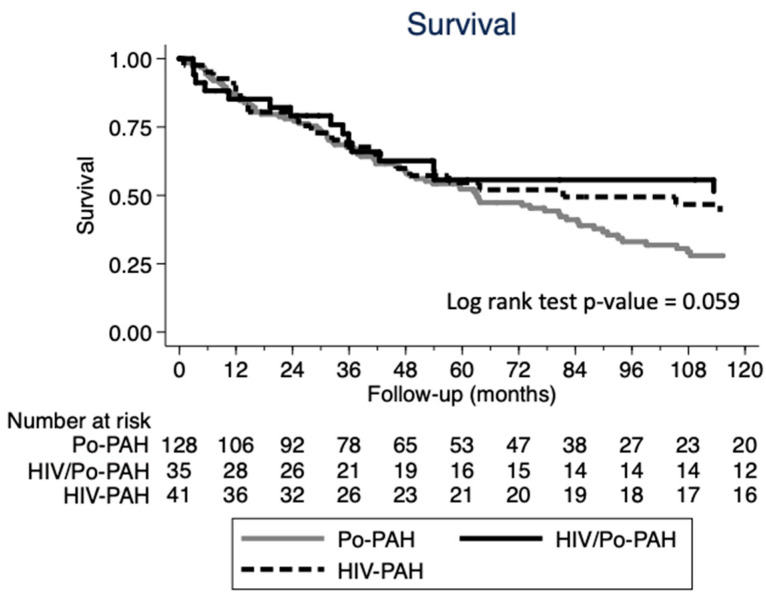
Kaplan–Meier curves showing the survival of the three groups of patients at 10 years.

The survival of the three study groups is shown in Figure 1.

## 4. Discussion

One of the aims of this study was to phenotype patients with PAH and overlapping HIV infection and portal hypertension, as they have never been extensively characterized despite representing, in our series, 21% of patients with PAH and portal hypertension and 46% of patients with PAH and HIV infection. This high percentage (higher than that observed in other case series reported in the literature) [15,24,25] is probably due to a higher percentage, in our patient population, of cases of portal hypertension associated with viral liver disease, of which HIV infection shares the transmission route and contagion risk factors. Another possible explanation can be related to the presence of a double trigger that may increase the risk of PAH, even if prospective cohort studies are needed to test this hypothesis.

In contrast to what has been described for other PAH etiologies, the proportion of men and women in patients with HIV/Po-PAH was similar. The same was true for patients with PAH and only HIV infection or portal hypertension. This feature has already been described in the literature [2,15,26,27,28,29] together with the lack of association of gender to the outcome of patients with PAH and portal hypertension and/or HIV [2,26,29].

As for the clinical, functional and hemodynamic profile and the exercise capacity, we observed that patients with HIV/Po-PAH had a better exercise performance as compared to patients with Po-PAH, probably because of their younger age [30]. There were no significant differences regarding liver function parameters, such as MELD-Na and CTP score, HVPG, and hemodynamic profile. Despite a comparable age, HIV/Po-PAH patients had a better exercise capacity as compared to HIV-PAH patients, probably due to a better hemodynamic profile (higher CI and SvO_2_ and lower PVR) that may be influenced by the hyperkinetic circulation related to portal hypertension. No differences in HAART prescription and CD4 count were observed. There were no significant differences between the three groups regarding baseline PAH risk profile, functional class, BNP/NT-proBNP levels, PAH treatment strategies and, as already analyzed, gender prevalence. 

As for PAH-specific treatment, despite the absence of a significant proportion of PAH patients with portal hypertension and/or HIV in the main randomized controlled trials for PAH therapies, our study population was intensively treated (>85% of our patients received PAH-targeted therapy). Combination therapy was started in the overall study period if PAH treatment goals were not met [20] or, in patients candidate to OLT, if transplant candidacy criteria were not met with only monotherapy. We observed that 60–70% of patients with Po-PAH maintained stable PAH severity profile on monotherapy throughout the follow-up (as in others cohorts described in the Literature) [15,28]. However, the survival of our study population was poor, with a 5-year survival of 51–55%. The discrepancy between the stability of the PAH disease and the poor outcome documented can be at least in part justified by the finding that the outcome was mainly related to the underlying disease rather than to PAH itself. 

It is well known that most patients with Po-PAH die because of complications related to the liver disease [15,31,32] and, in our cohort, the only independent predictors of survival in patients with Po-PAH were age and CTP score. This underlines the importance of a multidisciplinary approach in the management of patients with Po-PAH and, when OLT is deemed appropriate, the PAH treatment regimen must be appropriately tailored to obtain a permissive hemodynamic profile. 

Regarding HIV-PAH, we found that only HAART administration during follow-up was associated to a better outcome. Most studies have already demonstrated the beneficial effect of HAART on HIV-PAH patients outcome [33]. A possible explanation derives from the association between HIV-RNA plasma detection and the risk to develop PAH [34]. This has led to suggestions of a possible role for early HAART in reducing the risk of PAH progression [35]. However, considering that HAART has been associated to a mortality reduction both due to cardiac causes and infectious causes [36,37], and that HAART seems to be able to improve 6MWD but not hemodynamics in case of PAH [29], the results of our study can also be explained by the prognostic impact of HAART on HIV-related outcome and/or cardiac function improvement by HAART (rather than a direct effect on PAH-related outcome). Thus, in an appropriately treated PAH population, the prognosis of HIV infection may predominate over the prognosis related to the PAH itself. The latter hypothesis is also corroborated by the absence of a prognostic relevance of hemodynamic parameters in our cohort, in contrast with what has been documented by Degano et al. [29] who, however, included a good proportion of patients not receiving any PAH-specific drug. Moreover, unlike Degano et al., we did not find a prognostic role of baseline CD4 lymphocyte count, but this may be related to the lower percentage of patients receiving HAART at baseline in our study (60% vs. 81%) and to HIV treatment optimization changing the CD4 lymphocyte count during follow-up.

The main novelty of our study is the specific characterization of the population affected by PAH and dual pathogenetic triggers represented by HIV and portal hypertension. Regarding outcomes, we observed that their survival is similar to that of patients with only portal hypertension (who, however, have a non-statistically significant trend towards a worse prognosis, probably in relation to their older age that is, actually, one of their independent prognostic factors in our study) or HIV infection, despite the double pathogenetic noxa. Moreover, their survival was mainly related to the hepatic disease rather than to HIV infection, probably because HAART was prescribed in most of the patients and it is known that HAART can effectively reduce the mortality risk beyond improving the quality of life and the risk of opportunistic infections [38]. Instead, despite the improvements in the treatment of patients with portal hypertension, OLT frequently remains the only definitive treatment, but it may unfortunately be contraindicated by PAH itself or, in patients achieving a permissive hemodynamic profile, may be at least at an increased perioperative risk [39,40].

## 5. Limitations

The limitations of the paper include the retrospective analyses of a prospective registry as in all other studies on this topic. Moreover, we have not included in our evaluation data from other investigations such as echocardiography, MRI and cardiopulmonary exercise test because they were not systematically assessed. Eventually, we applied a simplified risk stratification tool that was proposed in our center only for patients with PAH and not externally validated; anyway, we also tested the COMPERA and the FPHR risk scores (supplementary Table S1), obtaining the same results.

## 6. Conclusions

Patients with HIV/Po-PAH are younger and with a better exercise capacity than patients with Po-PAH and have a better exercise capacity and hemodynamic profile compared to patients with HIV-PAH. Their prognosis is comparable to the two latter patient groups and seems to be related mainly to the underlying hepatic disease. Moreover, the prognosis of patients with Po-PAH and HIV-PAH seems to be mainly related to the underlying disease.

## Figures and Tables

**Table 1:** Baseline patient characteristics.

	Po-PAH	HIV/Po-PAH	HIV-PAH	*p*-Value
N	128	35	41	/
Male gender, *n* (%)	70 (55)	19 (54)	19 (46)	0.638
Age, years	53 (44–61) *^,∬^	44 (39–48) *	40 (37–44) ^∬^	**<0.001**
WHO-FC III-IV, *n* (%)	62 (48.4)–3 (2.3)	12 (34.3)–0	19 (46.3)–0	0.222
6MWD, m	426 (366–504) *	516 (432–571) *^,∔^	448 (370–510) ^∔^	**0.001**
Baseline risk, *n* (%)				0.063
- low	48 (37.5)	19 (54.3)	10 (24.4)	
- intermediate	70 (54.7)	15 (42.9)	25 (61.0)	
- high	10 (7.8)	1 (2.9)	6 (14.6)	
BNP/NT-proBNP, *n* (%)				0.488
- low	34 (46.0)	10 (62.5)	3 (37.5)	
- intermediate	26 (35.1)	3 (18.75)	2 (25.0)	
- high	14 (18.9)	3 (18.75)	3 (37.5)	
	(*n* tot = 74)	(*n* tot = 16)	(*n* tot = 8)	
PAH-specific therapy at baseline, *n* (%)				0.222
- None	116 (90.6)	34 (97.1)	37 (90.3)	
- Mono	3 (2.3)	0	3 (7.3)	
- Double	9 (7.1)	1 (2.9)	1 (2.4)	
PAH-specific therapy at the end of follow-up, *n* (%)				0.795
- None	19 (14.8)	5 (14.3)	6 (14.6)	
- Mono	77 (60.2)	25 (71.4)	26 (63.4)	
- Double	28 (21.9)	5 (14.3)	7 (17.1)	
- Triple	4 (3.1)	0 (0.0)	2 (4.9)	
Etiology of portal hypertension, *n* (%)				0.069
- Cirrhosis	112 (87.5)	34 (97.1)	/	
- Pre-hepatic	15 (11.7)	/	/	
- Post-hepatic	1 (0.8)	1 (2.9)	/	
MELD-Na	14 (11–18)	13 (10–15)	/	0.13
CTP, *n* (%)				
- A	68 (53.1)	21 (60.0)	/	0.768
- B	56 (43.8)	13 (37.1)	/	
- C	4 (3.1)	1 (2.9)	/	
HAART at baseline, *n* (%)	/	23 (66)	23 (56)	0.393
HAART at follow-up, *n* (%)	/	27 (77)	32 (78)	0.925
CD4, *n*/mmc	/	371 (200–530)	410 (294–535)	0.272
RAP, mmHg	6 (4–9)	6 (4–8)	8 (5–11)	0.105
mPAP, mmHg	45 (40–53)	45 (38–55)	48 (41–56)	0.272
PAWP, mmHg	8 (6–10)	7 (6–11)	7 (6–9)	0.147
mBP, mmHg	90 (80–98)	89 (83–104)	88 (78–97)	0.614
CI, L/min/m^2^	3.1 (2.6–3.8) ^∬^	3.0 (2.7–3.7) ^∔^	2.6 (2.2–2.9) ^∬,∔^	**<0.001**
PVR, WU	6.2 (4.9–8.8) ^∬^	7.0 (5.5–11.2) ^∔^	8.9 (7.5–12.2^)∬,∔^	**<0.001**
SVR, WU	14.0 (11.5–17.2) ^∬^	15.6 (12.8–18.9)	18.1 (15.7–21.6) ^∬^	**<0.001**
Syst O_2_ Sat, %	96 (95–97) ^∬^	97 (95–98) ^∔^	95 (94–96) ^∬,∔^	**0.011**
SvO_2_, %	70 (65–75) ^∬^	67 (62–71) ^∔^	61 (55–66) ^∬,∔^	**<0.001**
HVPG, mmHg	12 (8–16)	11 (8–17)	/	0.702

Legend: 6MWD = 6-min walk distance; BNP = brain natriuretic peptide; CI = cardiac index; CTP = Child–Turcotte–Pugh; HAART = highly active anti-retroviral therapy; HVPG = hepatic venous-portal gradient; mBP = mean blood pressure; MELD-Na = model for end-stage liver disease-Na; mPAP = mean pulmonary arterial pressure; NT-proBNP = N-terminal pro-brain natriuretic peptide; PAH = pulmonary arterial hypertension; PAWP = pulmonary artery wedge pressure; PVR = pulmonary vascular resistance; RAP = right atrial pressure; SvO_2_ = mixed venous oxygen saturation; SVR = systemic vascular resistance; Syst O_2_ Sat = systemic arterial oxygen saturation; WHO-FC = World Health Organization functional class. * ^∬ ∔^ = *p* < 0.05 between respective pairs.

**Table 2:** Univariate Cox regression analysis of Po-PAH patients.

	HR (95% CI)	*p*-Value
Male gender	1.193 (0.777–1.830)	0.420
Age, years	1.039 (1.019–1.059)	**<0.001**
Etiology of portal hypertension		
- Cirrhosis vs. Pre-hepatic	2.234 (1.029–4.848)	**0.042**
- Post-hepatic vs. Pre-Hepatic	12.386 (1.460–105.059)	**0.021**
MELD-Na	1.055 (1.013–1.098)	**0.010**
CTP, A/B/C	1.752 (1.216–2.524)	**0.003**
Baseline Risk, low/interm/high	1.367 (0.997–1.875)	0.052
WHO-FC III-IV	1.268 (0.831–1.935)	0.270
BNP/NT-proBNP, low/interm/high (*n* = 74)	1.235 (0.847–1.800)	0.272
6MWD, m	0.997 (0.995–0.999)	**0.011**
RAP, mmHg	1.005 (0.968–1.044)	0.787
mPAP, mmHg	0.982 (0.962–1.001)	0.068
PAWP, mmHg	0.974 (0.902–1.051)	0.500
mBP, mmHg	0.991 (0.975–1.007)	0.250
CI, L/min/m^2^	0.889 (0.685–1.154)	0.378
PVR, WU	1.000 (0.948–1.056)	0.987
SVR, WU	1.003 (0.965–1.042)	0.896
Syst O_2_ Sat, %	0.931 (0.861–1.007)	0.075
SvO_2_, %	0.994 (0.967–1.023)	0.685
HVPG, mmHg	1.042 (1.002–1.084)	**0.039**

Legend: 6MWD = 6-min walk distance; BNP = brain natriuretic peptide; CI = cardiac index; CTP = Child–Turcotte–Pugh; HVPG = hepatic venous-portal gradient; mBP = mean blood pressure; MELD-Na = model for end-stage liver disease-Na; mPAP = mean pulmonary arterial pressure; NT-proBNP = N-terminal pro-brain natriuretic peptide; PAH = pulmonary arterial hypertension; PAWP = pulmonary artery wedge pressure; PVR = pulmonary vascular resistance; RAP = right atrial pressure; SvO_2_ = mixed venous oxygen saturation; SVR = systemic vascular resistance; Syst O_2_ Sat = systemic arterial oxygen saturation; WHO-FC = World Health Organization functional class.

**Table 3:** Multivariate Cox regression analysis of Po-PAH patients.

	HR (95% CI)	*p*-Value
Age, years	1.039 (1.018–1.060)	<0.001
Child–Turcotte–Pugh, A/B/C	1.665 (1.153–2.403)	0.007

**Table 4:** Univariate Cox regression analysis of HIV-PAH patients.

	HR (95% CI)	*p*-Value
Male gender	1.366 (0.622–2.998)	0.437
Age, years	0.973 (0.908–1.043)	0.439
HAART at baseline	0.866 (0.394–1.904)	0.721
HAART during follow-up	0.283 (0.119–0.675)	**0.004**
CD4, *n*/mmc	0.999 (0.997–1.000)	0.155
Baseline risk, low/interm/high	1.864 (0.906–3.837)	0.091
WHO-FC III-IV	1.833 (0.829–4.051)	0.135
BNP/NT-proBNP, low/interm/high (*n* = 8)	1.878 (0.571–6.177)	0.299
6MWD, m	0.995 (0.991–0.999)	**0.006**
RAP, mmHg	1.069 (0.967–1.181)	0.192
mPAP, mmHg	1.019 (0.987–1.051)	0.253
PAWP, mmHg	1.035 (0.866–1.237)	0.706
mBP, mmHg	1.010 (0.976–1.046)	0.558
CI, L/min/m^2^	0.980 (0.583–1.647)	0.939
PVR, WU	1.081 (0.971–1.204)	0.154
SVR, WU	1.017 (0.920–1.123)	0.746
Syst O_2_ Sat, %	0.887 (0.793–0.992)	**0.036**
SvO_2_, %	0.983 (0.943–1.025)	0.422

Legend: 6MWD = 6-min walk distance; BNP = brain natriuretic peptide; CI = cardiac index; HAART = highly active anti-retroviral therapy; mBP = mean blood pressure; mPAP = mean pulmonary arterial pressure; NT-proBNP = N-terminal pro-brain natriuretic peptide; PAH = pulmonary arterial hypertension; PAWP = pulmonary artery wedge pressure; PVR = pulmonary vascular resistance; RAP = right atrial pressure; SvO_2_ = mixed venous oxygen saturation; SVR = systemic vascular resistance; Syst O_2_ Sat = systemic arterial oxygen saturation; WHO-FC = World Health Organization functional class.

**Table 5:** Univariate Cox regression analysis of HIV/Po-PAH patients.

	HR (95% CI)	*p*-Value
Male gender	1.072 (0.408–2.822)	0.887
Age, years	1.009 (0.942–1.082)	0.794
Etiology of portal hypertension		
- Post-hepatic vs. cirrhosis	7.931 (0.886–70.996)	0.064
MELD-Na	1.199 (1.044–1.376)	**0.010**
CTP, A/B/C	3.010 (1.203–7.532)	**0.019**
HAART at baseline	0.879 (0.334–2.317)	0.795
HAART during follow-up	0.433 (0.158–1.188)	0.104
CD4, *n*/mmc	0.999 (0.997–1.001)	0.253
Baseline risk, low/interm/high	0.485 (0.189–1.242)	0.131
WHO-FC III-IV	0.697 (0.245–1.985)	0.499
BNP/NT-proBNP low/interm/high (*n* = 16)	0.465 (0.155–1.395)	0.172
6MWD, m	1.000 (0.995–1.004)	0.827
RAP, mmHg	0.936 (0.811–1.080)	0.366
mPAP, mmHg	0.983 (0.941–1.028)	0.450
PAWP, mmHg	1.019 (0.868–1.196)	0.818
mBP, mmHg	0.948 (0.899–1.000)	0.051
CI, L/min/m^2^	2.032 (1.119–3.690)	**0.020**
PVR, WU	0.825 (0.689–0.988)	**0.037**
SVR, WU	0.826 (0.721–0.947)	**0.006**
Syst O_2_ Sat, %	1.117 (0.886–1.407)	0.350
SvO_2_, %	1.061 (0.985–1.142)	0.117
HVPG, mmHg	1.107 (1.011–1.213)	**0.029**

Legend: 6MWD = 6-min walk distance; BNP = brain natriuretic peptide; CI = cardiac index; CTP = Child–Turcotte–Pugh; HAART = highly active anti-retroviral therapy; HVPG = hepatic venous-portal gradient; mBP = mean blood pressure; MELD-Na = model for end-stage liver disease-Na; mPAP = mean pulmonary arterial pressure; NT-proBNP = N-terminal pro-brain natriuretic peptide; PAH = pulmonary arterial hypertension; PAWP = pulmonary artery wedge pressure; PVR = pulmonary vascular resistance; RAP = right atrial pressure; SvO_2_ = mixed venous oxygen saturation; SVR = systemic vascular resistance; Syst O_2_ Sat = systemic arterial oxygen saturation; WHO-FC = World Health Organization functional class.

**Table 6:** Multivariate Cox regression analysis of HIV/Po-PAH patients.

	HR (95% CI)	*p*-Value
MELD-Na	1.224 (1.064–1.408)	0.005
HVPG, mmHg	1.125 (1.026–1.235)	0.013

Legend: HVPG = hepatic venous-portal gradient; MELD-Na = Model for End-Stage Liver Disease-Na.

## Data Availability

The data supporting the findings of this study are available from the corresponding author upon reasonable request.

## Supplementary Materials

The following supporting information can be downloaded at: https://www.mdpi.com/article/10.3390/jcm12103425/s1, Table S1: Univariate Cox regression analysis of Comparative, Prospective Registry of Newly Initiated Therapies for Pulmonary Hyper-tension (COMPERA) and French Pulmonary Hypertension Registry (FPHR) risk scores in the 3 study groups.
