# Supplementary material for: Pulmonary Arterial Hypertension Associated with Portal Hypertension and HIV Infection: Comparative Characteristics and Prognostic Predictors
Source: J Clin Med. 2023 May 12;12(10):3425. doi: 10.3390/jcm12103425 (PMC10219491; doi:10.3390/jcm12103425)
Supplement: Supplementary file 1 [file jcm-12-03425-s001.zip › jcm-2371615-supplementary.pdf]

Supplementary Table S1. Univariate Cox regression analysis of Comparative, Prospective Registry of Newly Initiated Therapies for Pulmonary Hyper-tension (COMPERA) and French Pulmonary Hypertension Registry (FPHR) risk scores in the 3 study groups.

	**HR (95% CI)**	**p-value**
- Po-PAH		
COMPERA baseline risk, low/interm/high	1.390 (0.948 – 2.037)	0.091
FPHR baseline risk, low/interm/high	1.365 (0.978 – 1.903)	0.067
- HIV-PAH		
COMPERA baseline risk, low/interm/high	1.723 (0.843 – 3.519)	0.136
FPHR baseline risk, low/interm/high	1.397 (0.805 – 2.425)	0.234
- HIV/Po-PAH		
COMPERA baseline risk, low/interm/high	0.481 (0.156 – 1.483)	0.203
FPHR baseline risk, low/interm/high	0.546 (0.157 – 1.902)	0.342
